# CBD attenuates amygdala response to negative emotional stimuli in individuals with alcohol use disorder – a randomized controlled trial

**DOI:** 10.1007/s00213-025-06860-5

**Published:** 2025-08-29

**Authors:** Marlen Pfisterer, Anton Teetzmann, Sina Vetter, Joscha Baeßler, Lena Schreckenberger, Judith Zaiser, Manuel Stenger, Patrick Bach

**Affiliations:** 1 Department of Addictive Behavior and Addiction Medicine, Central Institute of Mental Health, Medical Faculty Mannheim/Heidelberg University, J5/68159 Mannheim, Germany; 2 German Center for Mental Health (DZPG), Partner Site Mannheim-Heidelberg-Ulm, Mannheim, Germany; 3 Feuerlein Center On Translational Addiction Medicine (FCTS), University of Heidelberg, Heidelberg, Germany; 4 Department of Clinical Neuroscience, Karolinska Institutet, Stockholm, Sweden

**Keywords:** FMRI, Cannabidiol, Alcohol Use Disorder, Faces, Emotion, Craving

## Abstract

**Rationale:**

Negative affect is a key factor in Alcohol Use Disorder (AUD) associated with craving and relapse risk that is insufficiently treated by approved medications. Cannabidiol (CBD) has shown promising effects on negative affect, indicating its potential for addressing the neurocircuitry underlying negative affect in AUD.

**Objectives:**

This study investigates CBD’s effects on neural response to negative emotional stimuli and subjective alcohol craving in individuals with AUD.

**Methods:**

We conducted the first neuroimaging study investigating CBD’s effects on neural responses to negative emotional stimuli and craving in AUD. The study was designed as two armed, 1:1 randomized, double blind, parallel group neuroimaging trial, enrolled *N* = 28 individuals with AUD. It compared the effects of 800 mg oral CBD versus matched Placebo (PLC) on blood oxygenated level dependent (BOLD) response in the amygdala during a validated emotion processing task and explored associations with CBD plasma levels and subjective alcohol craving.

**Results:**

CBD versus PLC attenuated bilateral amygdala reactivity to angry and fearful faces (*p*_FWE_ <.05, small volume corrected), while CBD showed no effect on amygdala activation during the presentation of neutral shape stimuli. Amygdala response to negative emotional stimuli correlated positively with the extent of subjective alcohol craving (*r*_Left Amygdala_ =.52, *p*_FDR_ =.01; *r*_Right Amygdala_ =.52, *p*_FDR_ =.01) and negatively with CBD plasma levels (*r*_Left Amygdala_ = -.68, *p*_FDR_ = 0.002; *r*_Right Amygdala_ = -.65, *p*_FDR_ = 0.002).

**Conclusion:**

In summary, CBD’s effects on amygdala reactivity to negative emotional stimuli in individuals with AUD support CBD’s potential for modulating emotion-processing circuits in AUD and CBD’s treatment potential for craving and relapse driven by negative affective states.

**Supplementary Information:**

The online version contains supplementary material available at 10.1007/s00213-025-06860-5.

## Introduction

Alcohol use disorder (AUD) affects an estimated number of 400 million people worldwide and is a major risk factor for death and disability (WHO 2024). High prevalence of AUD contrasts with insufficient treatment outcomes with high relapse rates. This stresses the need to enhance and advance pharmacological AUD treatments (Litten et al. 2018; Köhne 2024; Heilig, et al. 2024). In the development and maintenance of AUD, negative affect plays a pivotal role (Dean et al. 2020). However, treatment options targeting exacerbated negative affect in AUD are still lacking. The amygdala is a core substrate of emotion processing that demonstrated associations with relapse risk in AUD (Koob and Volkow 2010; See et al. 2003). It plays a critical role in cue-associated learning, cue-induced craving and relapse to drug-seeking behavior (Luo et al. 2013). Therefore, medications that modulate emotion processing and amygdala activity might be promising for treating AUD.

In this context, Cannabidiol (CBD), a non-psychoactive compound derived from the cannabis plant, attracted increasing attention (Turna et al. 2019; Weiss and Gonzalez-Cuevas 2019). CBD influences neurotransmitter pathways and alters functional connectivity within brain regions associated with AUD (Hurzeler et al. 2024). It acts as a negative allosteric modulator of the cannabinoid- (CB1) receptor and regulates CB1 receptor activation (Laprairie et al. 2015). CB1 receptors are densely localized in amygdala, a brain structure which has been implicated in alcohol craving, drug seeking and emotion processing (Katona et al. 2001; Wirz et al. 2018). Previous studies highlighted the stress-reducing, anxiolytic and anti-depressive effects of CBD in animal models of AUD (Iffland and Grotenhermen 2017; Viudez-Martínez et al. 2018; Gom 2024). Emerging clinical data suggests that CBD holds promise as potential therapeutic agent for substance use disorders (SUDs) (Paulus, et al. 2022), potentially via its effects on substance use driven by stress and negative affect. This is supported by a clinical trial in individuals with opioid use disorder, which showed that a single dose of CBD significantly reduced opioid craving and anxiety (Hurd et al. 2019). Effects of CBD on negative affective states were also indicated by studies in anxiety, psychotic disorders and depression (García-Gutiérrez, et al. 2020). Here, CBD has been shown to attenuate anxiety after exposure to social stress in healthy individuals (Zuardi et al. 1993; Zuardi et al. 2017). Anxiolytic effects have also been reported in patients with social anxiety disorder (Fliegel and Lichenstein 2022), while, to date, clinical evidence for CBD’s effects on depressive symptoms remains limited (Hurd et al. 2019; Bilbao and Spanagel 2022). In line with these findings, there is a overall growing interest in the effects of CBD on negative affect states in SUDs. However, in the context of AUD, specific effects of CBD on the neural circuits implicated in the processing of negative emotions, particularly within the amygdala, remain underexplored. Addressing this vacuum as scientific desideratum, the here presented study was designed as first proof-of-principle experimental neuroimaging study to investigate the effects of CBD on amygdala activation during emotion processing in AUD. The study compared the effects of a single dose of 800 mg oral CBD against matched oral PLC on amygdala activation during a validated face-matching functional magnetic resonance imaging (fMRI) task and explored associations between amygdala activation, subjective alcohol craving and CBD plasma levels.

## Methods and materials

The ICONIC trial as a whole was designed to investigate the effects of CBD compared to PLC on experimental models for two central stages of the addiction cycle and subjective alcohol craving, specifically amygdala response to emotional stimuli (as an experimental model for the negative affect/withdrawal state (Koob and Volkow 2010)) and striatal alcohol cue-reactivity (as an experimental model for the binge/intoxication state (Koob and Volkow 2010)) that were chosen, due to their close links with alcohol craving and clinical outcomes. Here, we focus on the analyses of CBD’s effects on emotion processing and explore the associations with alcohol craving and CBD plasma levels.

### Study design

The trial was designed as a two armed, 1:1 randomized, double blind, parallel group neuroimaging study. It was carried out at the Central Institute of Mental Health (CIMH) in Mannheim, Germany. The trial was preregistered (German clinical trials database: DRKS00029993, study protocol can be accessed via DRKS.de; date of registration: 2022–08-10). All experimental procedures were approved by the local ethics committee of the University of Heidelberg. The clinical trial was designed and conducted in compliance with Good Clinical Practices (ICH-GCP) and the Declaration of Helsinki.

### Study sample

The study enrolled 28 non-treatment seeking individuals with AUD according to the Diagnostic and Statistical Manual of Mental Disorders DMS-V of both sexes. Participants were recruited from the general public via advertisements. They were instructed to remain abstinent at least 24 h before the beginning of the test session. Individuals were excluded from the trial, if they showed any withdrawal symptoms, positive breath alcohol test or positive drug screening at the time of the experimental assessment visit. Further exclusion criteria comprised current psychotic or bipolar disorders or current severe depressive episodes, acute suicidal tendencies, contraindications for functional magnetic resonance imaging, pregnancy, lactation or breastfeeding, current severe somatic comorbidities or a hypersensitivity to CBD or to any drug with similar chemical structure. Detailed information on the sample size estimations is provided in the Supplementary Material (see Supplements).

### Study procedures

The trial consisted of two on-site visits, which were scheduled on two consecutive days, as screening visit and assessment visit. In the screening visit, participants underwent an assessment of individual characteristics such as substance use, psychiatric and somatic comorbidities to evaluate inclusion/exclusion criteria and written informed consent was obtained. To ensure the anonymity of all participants, subjects were identified solely by means of their pseudonymized individual identification code during the study. Individuals who did not consent to circulate their pseudonymised data were not included into the study. The data obtained in the course of the study was treated in accordance to the EU General Data Protection Regulation (GDPR) and national regulatory requirements (e.g. Federal Data Protection Act).

Participants that consented to participate in the study were invited to a following assessment visit which started with a breath alcohol test, a drug urine and a pregnancy test. It comprised multiple self-report questionnaires on alcohol craving (Alcohol Urge Questionnaire: AUQ (Bohn et al. 1995)) and on affective symptoms (Primary Appraisal Secondary Appraisal Questionnaire: PASA (Gaab and PASA 2009); Positive Affect Negative Affect Schedule: PANAS (Watson et al. 1988). Beck Depression Inventory (revised version): BDI-II (Hautzinger et al. 2006)). Participants were then randomized to receiving either 800 mg CBD as four tablets of 200 mg Cannabidiol (pure CBD with THC content < 0.5%, provided by Endosane Pharmaceuticals, Germany) or matched oral placebo tablets with identical ingredients except for CBD (provided by Endosane Pharmaceuticals, Germany). The independent pharmacy of Heidelberg University Hospital generated the block randomization schedule, maintaining blindings until final participant assessment. Appearances of CBD and PLC containers were identical and only specified by pseudonymized individual identification code. Safety studies showed that CBD is overall well tolerated with only mild drug side effects (Chesney et al. 2020; Larsen and Shahinas 2020). Most commonly reported adverse effects were tiredness, diarrhea, or changes in appetite (Iffland and Grotenhermen 2017). CBD dosage was determined based on previous studies that demonstrated a dose–response relationship, showing greater efficacy of an 800 mg CBD dosage compared to lower doses in reducing drug craving (Martinez Naya et al. 2024; Sholler et al. 2020). For this dosage, previous research also demonstrated significant effects on cerebral blood flow in the amygdala, which was the region of interest of the presented analyses (Hurd et al. 2019; Crippa et al. 2004). CBD or PLC was administered 180 min before the combined stress- and cue-exposure and the fMRI assessment, due to an estimated peak plasma and brain concentration of CBD 120 to 360 min after oral administration and in line with previous studies (Hurd et al. 2019). The 800 mg fixed dose corresponded to an average weight-adjusted dose of 10.7 (SD 2.2, range: 6.9–15.4) mg/kg CBD. Plasma samples were drawn before the fMRI sessions, to determine CBD levels (see Supplements for details). CBD plasma concentrations were determined to explore potential associations between CBD plasma levels and both neural and behavioral outcomes. Assessing plasma levels appeared to be particularly relevant in the context of oral CBD administration, as its bioavailability can vary across individuals (Davies et al. 2020). Thus, CBD plasma levels were determined in order to enable a more precise interpretation of potential dose-dependent effects on subjective alcohol craving and neural responses during fMRI assessments.

The fMRI assessment included paradigms assessing emotion processing (Hariri et al. 2002), neural alcohol cue-reactivity and subjective alcohol craving (Vollstadt-Klein et al. 2012), response to natural rewarding stimuli (Zimmermann et al. 2022) and structural scans. According to the preregistration of the study, the effect of CBD on neural alcohol cue-reactivity in the Nucleus accumbens of individuals with alcohol use disorder was predefined as primary outcome, measured using the blood oxygenated level dependent (BOLD) response. The case number was determined based on the power calculation for the primary endpoint of the main study. Detailed information on the sample size estimation is provided in the Supplementary Material (see Supplements). Secondary outcomes comprised the effects of CBD vs. PLC administration on neural brain activation during the presentation of emotional faces and neutral shapes, measured using the BOLD response. The presented study focused on the investigation of the effects of CBD on neural response to negative affective stimuli during an fMRI emotion-processing task as preregistered secondary outcome and its associations with subjective alcohol craving assessed during the alcohol cue-reactivity paradigm. Results of data about subjective alcohol craving during the fMRI cue-reactivity paradigm were used to assess associations between amygdala activation, subjective alcohol craving and CBD plasma levels. Detailed results on the effects of CBD on brain activation during the presentation of alcohol cues within the framework of the alcohol cue-reactivity task are not reported here, as these results have been published separately (Zimmermann et al. 2024). In short, these prior analyses on the effects of CBD on brain activation during the presentation of alcohol cues showed that CBD attenuated alcohol cue-induced brain activation in the bilateral Nucleus accumbens (NAc) and alcohol craving. In addition, the magnitude of CBD’s effects on both outcomes was positively correlated with CBD plasma levels during the experiment, indicating that CBD attenuated NAc activation and craving in a plasma-level dependent manner (Zimmermann et al. 2024).

### Assessment of neural response to negative emotional stimuli and assessment of alcohol craving

We used a validated face-matching task to investigate emotion processing (Hariri et al. 2002; García-García et al. 2016). The task contained two categories of images, either angry or fearful facial expressions or neutral geometric shapes. Each image was shown for two seconds, with an inter-stimulus-interval of one second. Stimuli were presented in blocks of six stimuli of the same category, with the entire task consisting of eight blocks. Of these, four blocks were allocated to geometric shapes and four blocks to emotional facial expressions. The blocks of emotional facial expressions were equally divided between angry and fearful expressions (i.e. two blocks each) to ensure a balanced presentation. The task took a total of eight minutes. Participants were instructed to identify which of the two inferior images matched the image at the top. According to their choices, they pressed a button corresponding to the image on the left versus on the right. During the task, reaction time and correctness of button presses (correct versus incorrect) were recorded. Subjective alcohol craving was assessed during the alcohol stimulus reactivity paradigm, during which blocks of alcohol associated images and of neutral images were presented to the participants. Participants were asked to indicate their current alcohol craving on a visual analog scale (VAS) ranging from zero (no craving at all) to 100 (very intense craving). The entire alcohol stimulus reactivity paradigm consisted of 21 image blocks presented over approximately 12 min. 12 blocks included alcohol-associated images, and nine blocks included neutral control images. Each block contained five stimuli, resulting in a total of 105 images (for a detailed description, please refer to Zimmermann et al. (2024)).

#### MRI data acquisition and pre-processing

MRI data was captured by a three Tesla whole-body-tomograph MAGNETOM 3 Tesla whole-body-tomograph (MAGNETOM PRIMSA^fit^, Siemens, Erlangen, Germany). A total of 315 T2*-weighted echo-planar images (EPI) were acquired during the validated face-matching task to investigate emotion processing using the CMRR multi-band EPI sequence (Setsompop et al. 2012; Moeller et al. 2010) (TR = 0.869 s, TE = 38 ms, flip angle = 58°, 60 interleaved slices, slice thickness = 2.4 mm, voxel dimensions = 2.4 × 2.4x2.4 mm^3^, FOV = 210 × 210mm^2^, 88 × 88 matrix, AP phase-encoding, multi-band factor 6, bandwidth 1832 Hz/Px, MB LeakBlock Kernel, weak raw filter, prescan normalization, excite pulse duration 7 ms). Field map images were acquired with a standard Siemens dual gradient echo sequence (TR = 0.698 s, TE1 = 5.19 ms, TE2 = 7.65 ms, flip angle = 54°, 64 interleaved slices, slice thickness = 2.4 mm, voxel dimensions 2.4 × 2.4x2.4 mm^3^, FOV = 210 × 210mm^2^, 88 × 88 matrix, AP phase-encoding, bandwidth 279 Hz/Px).

In order to reduce artefacts due to magnetic saturation effects, the first five scans were excluded from analyses. All MRI data were pre-processed using the statistical parametric mapping software for Matlab (SPM, Wellcome Department of Cognitive Neurology, London, UK) version 12. Image data were temporally realigned to minimize differences in slice acquisition, corrected for residual geometric distortion on the basis of the acquired magnetic field maps, spatially realigned, corrected for micro-movements and normalized to a standard MNI [Montreal Neurological Institute, Quebec, Canada] EPI template implemented in SPM12. Subsequently, images were smoothed using an isotropic Gaussian kernel for group analysis [8 mm Full Width at Half Maximum]). Motion during fMRI was assessed through visual inspection of the realignment parameters. Movement was considered excessive with > 2 mm translation in any direction or > 2° rotation in any direction.

For every participant, first level statistics were computed, modelling the different experimental conditions (faces, shapes) in a general linear model (GLM) and adding movement parameters as nuisance variables in the GLM, according to the SPM manual and previous studies on this paradigm (Kirsch et al. 2005). Resulting contrast images for the “faces versus shapes” were imputed in the following second-level analyses.

### MRI data analysis

A total of 28 participants were enrolled in the study of which 25 participants provided fMRI data for the presented analyses (n_CBD_group_ = 12, n_PLC_group_ = 13). The primary analysis included all randomized patients with fMRI data (N = 25). A one-sample t-test was used to assess differences between neural responses during the presentation of emotional face stimuli versus neutral geometric shapes. The primary analysis compared the left and right amygdala response to emotional face stimuli between both treatment groups using a two-sample t-test in the SPM12 software. In accordance to our strong a priori hypothesis for the amygdala, we conducted region-of-interest (ROI)-based analyses for this region using the small volume correction (SVC) function of SPM12 that restricted the search area to the standardized anatomical left and right amygdala masks from the Wake Forest University PickAtlas (WFU PickAtlas: http://www.fmri.wfubmc.edu/downloads). We applied a cluster-size threshold within the volume of interest of *p*_FWE_ < 0.05 (two ROI analyses in total).

In order to explore associations between amygdala activation, alcohol craving and CBD plasma levels, we extracted the mean brain activation in the left and right amygdala for the different task contrasts (contrasts: “faces versus implicit baseline”, “shapes versus implicit baseline” and “faces versus shapes”). To this end, we used the standard functions included in the MarsBar software package (https://marsbar-toolbox.github.io/index.html) and standard anatomical masks for the left and right amygdala from the WFU PickAtlas to extract mean brain activation. These data were exported to IBM SPSS Statistics version 29.0 for following analyses. Associations of functional brain activation in the left and right amygdala ROIs with alcohol and CBD plasma levels were tested using Pearson correlation coefficient and corrected for multiple testing using the False Discovery Rate (FDR) correction procedure and bootstrapping, using the Bias corrected and accelerated (BCa) bootstrapping procedure as implemented in IBM SPSS version 29.0 with 1000 random samples.

## Results

### Sample characteristics

A total of 25 individuals with AUD were included in the primary analysis. The first participant was recruited on 17/01/2023. The trial ended on 28/09/2023, when the last participant was enrolled. Participants were on average 34.7 years old (*SD* = 12.2) and met on average 5.1 (*SD* = 2.0) AUD criteria, which is equivalent to moderate AUD. They reported an average alcohol consumption of 43 g alcohol per day (*SD* = 24.7). Participants reported alcohol consumption on 50% of all days (*SD* = 0.2) and heavy drinking on 40% of all days (*SD* = 0.2). Participants reported last alcohol consumption on average approximately 3 days prior to the study visit, with no significant difference between CBD and PLC group (CBD *M* = 2.77, *SD* = 4.75; PLC *M* = 3.75, *SD* = 1.79), as confirmed by an independent samples t-test (*t*(23) = −0.731, *p* = 0.472). All participants were abstinent from alcohol and drugs at the time of assessment, confirmed by a drug urine screening and breath alcohol test. Participants of both groups did not differ on any demographical variable, substance use indices or psychometric variables (Table 1).

**Table 1:** Baseline data on demographic characteristics, alcohol use and severity measures for participants randomized to the cannabidiol and placebo treatment arms with available neuroimaging data (*N* = 25)

	1	2		
	CBD (*n* = 12)	PLC (*n* = 13)	Statistics	Significance
Demographical variables (self-reported)				
Sex^A^ [male/female; number (%)]	9 (75%)/3 (25%)	8 (62%)/5 (38%)	Z = 0.52	*p* = 0.67
Age [years; mean (SD)]	36.75 (7.38)	32.85 (15.43)	*t*(17.51) = 0.82	*p* = 0.43
Race/ethnicity				
White [number (%)]	12 (100%)	13 (100%)	-	-
European ancestry [number (%)]	12 (100%)	13 (100%)	-	-
Substance use				
AUD criteria [sum; mean (SD)]	4.75 (1.87)	5.38 (2.22)	*t*_(23)_ = 0.77	*p* = 0.45
Audit [total score; mean (SD)]	7.33 (1.61)	7.00 (2.20)	*t*_(23)_ = 0.43	*p* = 0.67
ADS [total score; mean (SD)]	15.33 (5.93)	12.38 (4.94)	*t*_(23)_ = 1.36	*p* = 0.19
Days since last alcohol use [mean (SD)]	3.75 (4.28)	2.77 (1.72)	*t*_(23)_ = 0.76	*p* = 0.47
Mean daily alcohol use last 90 days [gram/day; mean (SD)]	37.06 (15.71)	48.39 (30.43)	*t*_(23)_ = 1.15	*p* = 0.26
Percent heavy drinking days last 90 days [gram/day; mean (SD)]	0.27 (0.14)	0.41 (0.29)	*t*_(23)_) = 1.48	*p* = 0.15
CBD use lifetime [yes/no; number (%)]	4 (33%)/8 (67%)	6 (46%)/7 (54%)	Z = 0.43	*p* = 0.69
CBD use last 30 days [yes/no; number (%)]	1 (8%)/11 (92%)	2 (15%)/11 (85%)	Z = 0.29	*p* = 1.00
THC use lifetime [yes/no; number (%)]	10 (83%)/2 (17%)	12 (92%)/1 (8%)	Z = 0.48	*p* = 0.59
THC use last 30 days [yes/no; number (%)]	3 (25%)/9 (75%)	1 (8%)/12 (92%)	Z = 1.39	*p* = 0.32
Current cigarette smoker [yes/no; number (%)]	3 (25%)/9 (75%)	3 (23%)/10 (77%)	Z = 0.01	*p* = 1.00
Psychometric data^a^				
AUQ at baseline (T0) [total score; mean (SD)]	15.58 (5.45)	15.69 (6.21)	*t*(23) = 0.46	*p* = 0.96
BDI [total score; mean (SD)]	13.58 (8.21)	18.08 (12.22)	*t*(23) = 1.07	*p* = 0.30
STAI trait [total score; mean (SD)]	43.42 (8.48)	49.85 (10.43)	*t*(23) = 1.68	*p* = 0.11
PANAS positive affect [total score; mean (SD)]	32.17 (7.74)	25.85 (9.92)	*t*(23) = 0.21	*p* = 0.84
PANAS negative affect [total score; mean (SD)]	25.08 (10.98)	25.85 (9.92)	*t*(23) = 0.18	*p* = 0.86
PASA [stress index score; mean (SD)]	−2.18 (0.99)	−1.94 (1.57)	*t*(23^B^) = 0.44	*p* = 0.66
CBD level (ng/ml)	290.63 (245.31)	0.36(0.20)	*t*(11^B^) = −4.10	*p* = 0.002
Emotion-processing task				
Reaction times face trials (ms)	1317.25	1259.71	*t*(23) = −0.447	*p* = 0.659
Reaction times shape trials (ms)	1235.51	1227.20	*t*(23) = −0.082	*p* = 0.935
Correct responses face trials (%)	98.96	100	*t(*23) = 1.043	*p* = 0.308
Correct responses shape trial in %	94.79	96.79	*t*(23) = 0.752	*p* = 0.459

*AUD* Alcohol Use Disorder, *AUDIT* Alcohol Use Disorders Identification Test, *ADS* Alcohol Dependence Scale, *AUQ* Alcohol Urge Questionnaire, *BDI* Beck Depression Inventory, *STAI* State Trait Anxiety Inventory, *PANAS* Positive and Negative Affect Schedule, PASA = Primary Appraisal Secondary Appraisal;

^A^Gender and sex were recorded by self-report and were consistent with each other;

^B^adjusted degrees of freedom according to standard procedures implemented in IBM SPSS version 29.0, due to unequal variances, indicated by positive Levene test

### Brain activation

**Fig. 1:**
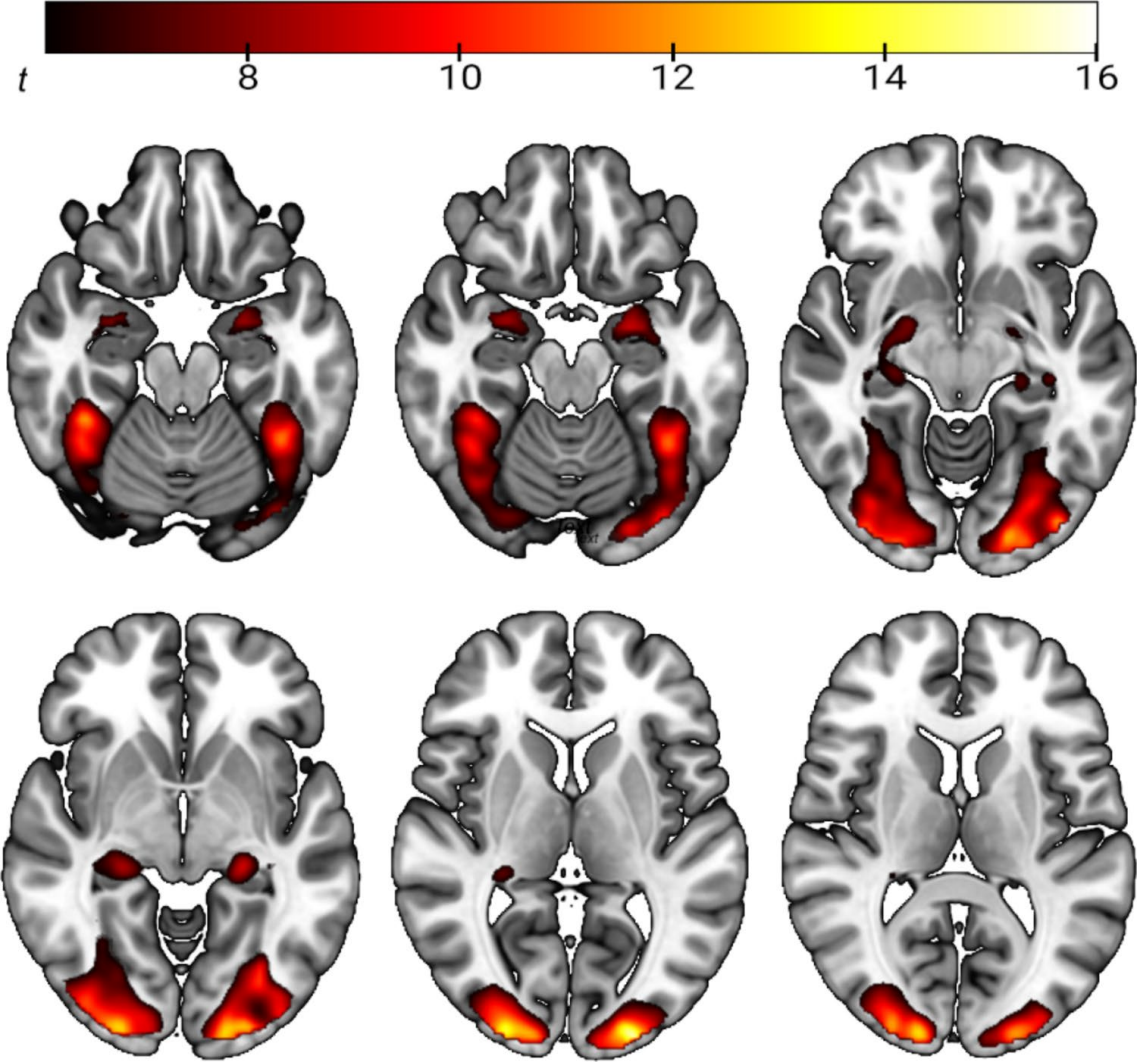
Depiction of brain areas that show higher activation during presentation of emotional face expressions versus geometric shapes in the whole sample (one-sample t-test, contrast: “faces– shapes”, p_*FWE*_ < 0.05 whole-brain corrected)

**Fig. 2:**
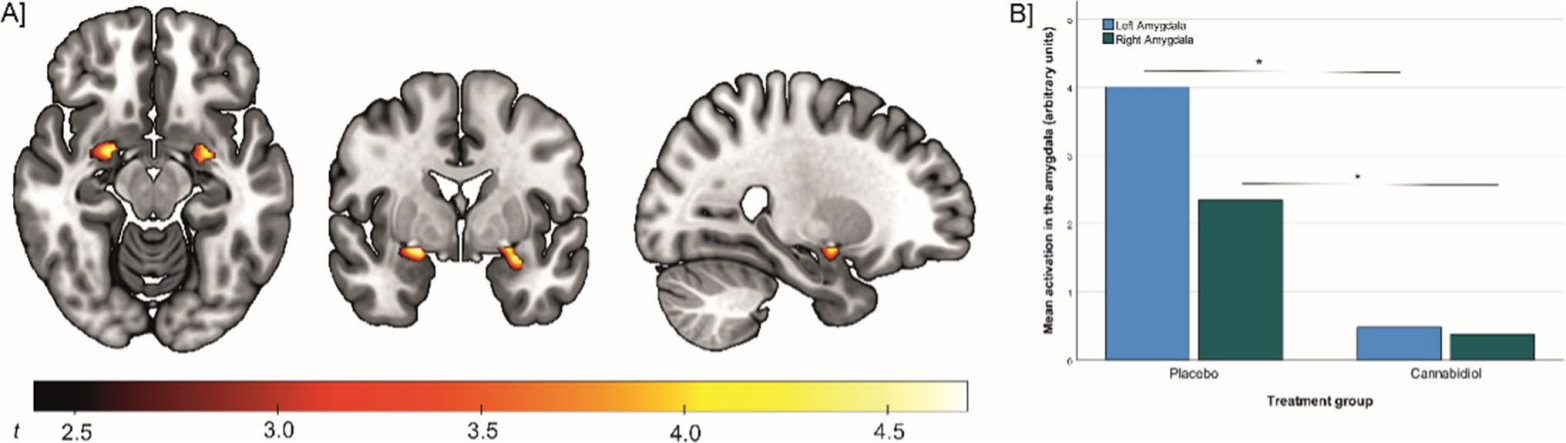
Depiction of lower amygdala activation in individuals treated with 800 mg CBD, compared to participants treated with placebo, during the presentation of emotional face expressions versus geometric shapes (two-sample t-test, CBD group < Placebo group; contrast: “faces – shapes”, p_*FWE*_ < 0.05 small volume corrected)

Whole-brain-analysis showed that face stimuli, compared to geometric shapes, induced higher brain activation in the bilateral amygdalae and the hippocampus, thalamus, fusiform gyrus and parts of the parietal, occipital and temporal gyri (see Fig. 1 and Supplementary Table S1, see Supplements). The comparison between both treatment groups evidences that CBD-treated individuals had a lower activation in the left (*t*_(23)_ = 4.80, cluster size = 58 voxel, *p*_FWE cluster level_ = 0.004, MNI peak coordinate X/Y/Z = −28/−2/−16) and right amygdala (*t*_(23)_ = 4.75, cluster size = 35 voxel, *p*_FWE cluster level_ = 0.007, MNI peak coordinate X/Y/Z = 22/−2/−12) during the presentation of emotional face stimuli than individuals receiving placebo (see Fig. 2). Additional whole-brain analysis indicated no significant effects of CBD treatment on brain activation in other brain areas. Further, analysis of amygdala activation during the presentation of emotional faces (versus implicit baseline) and shapes (versus implicit baseline) indicated that the treatment effect was specific to the emotion-processing condition of the task. Specifically, there was a significant lower activation in left and right amygdala in the CBD-treated group, compared to the PLC group, during the presentation of emotional face stimuli (versus implicit baseline) (*t*_(23)left Amgydala_ = 17,188, *p* < 0.001, d = 0.48; *t*_(23)right Amygdala_ = 11.640, *p* < 0.001, d = 0.44), but not during presentation of neutral geometric shapes (versus implicit baseline) (*t*_(23)left Amgydala_ = −1.222, *p* = 0.234, d = 0.45; *t*_(23)right Amygdala_ = 0.636, *p* = 0.531, d = 0.42, see Supplementary Fig. S1).

### Associations between amygdala activation, alcohol craving and CBD plasma levels

**Fig. 3:**
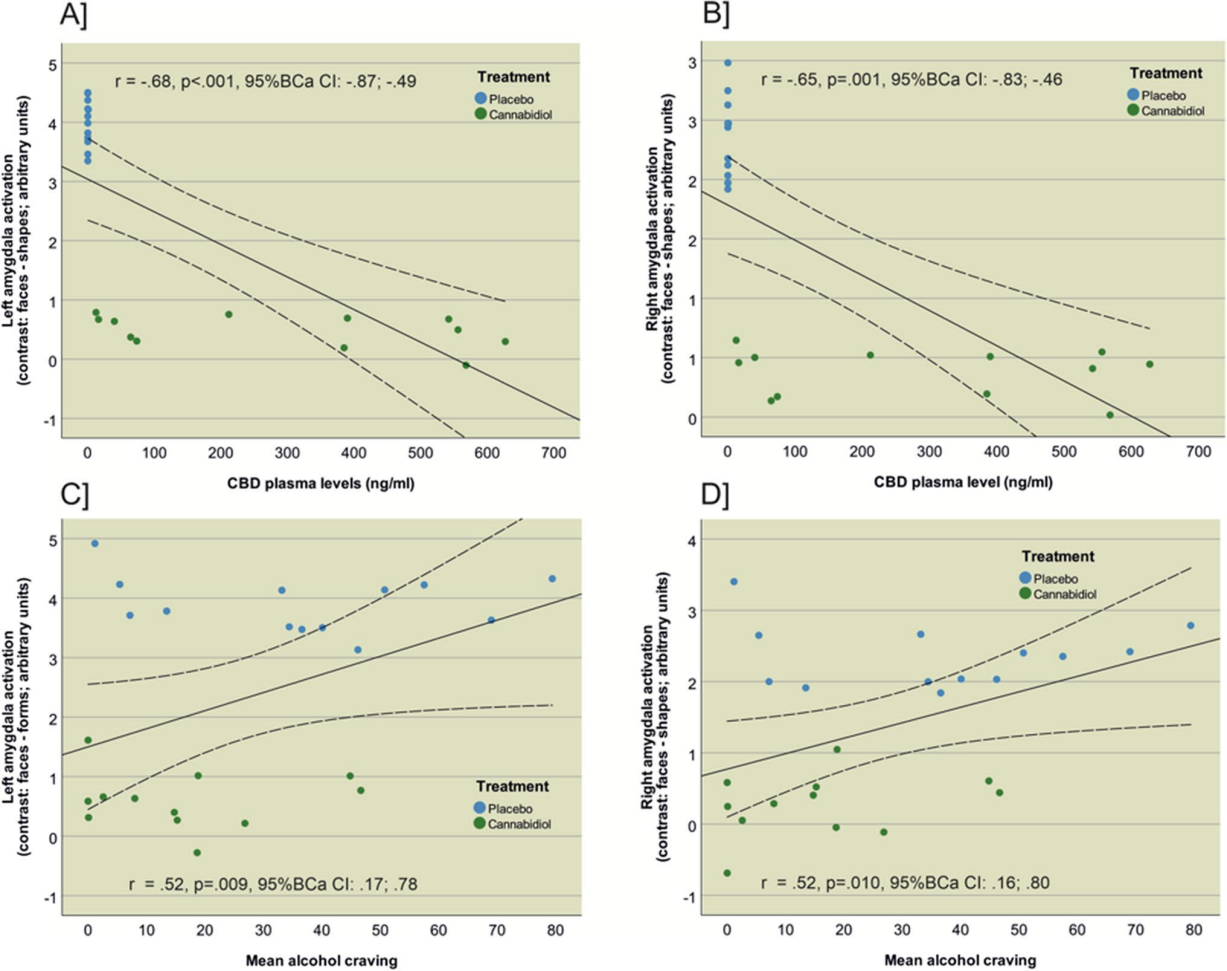
Depiction of the significant negative correlation between **A**) left amygdala activation and mean plasma level (ng/ml) (r = −0.68, Bias corrected and accelerated 95% confidence interval [95% BCa CI] = −0.87;−0.49, *p* < 0.001, p_FDR_ = 0.002); **B**) right amygdala activation and mean plasma level (ng/ml) (r = −0.65, Bias corrected and accelerated 95% confidence interval [95% BCa CI] = −0.83;−46, *p* = 0.001, p_FDR_ = 0.002; **C**) left amygdala activation and mean alcohol craving (r = 0.52, Bias corrected and accelerated 95% confidence interval [95% BCa CI] = 0.17;0.78, p = 0.009, p_FDR_ = 0.01); **D**) right amygdala activation and mean alcohol craving (r = −0.52, Bias corrected and accelerated 95% confidence interval [95% BCa CI] = 0.16;0.80, p = 0.010, p_FDR_ = 0.01)

The analysis revealed significant associations between amygdala activation, mean CBD plasma levels and alcohol craving (see Fig. 3). The analysis showed a significant negative correlation between left amygdala activation (contrast: face – shape) and mean CBD plasma levels (*r*_Left Amygdala_ = −0.68, *p* < 0.001, *p*_FDR_ = 0.002, Bias corrected and accelerated 95% confidence interval [95% BCa CI] = −0.87 to −0.49). Right amygdala activation was also negatively correlated with mean CBD plasma levels (*r*_Right Amygdala_ = −0.65, *p* = 0.001, *p*_FDR_ = 0.002, 95% BCa CI = −0.83 to −0.46), indicating that higher mean CBD plasma levels were associated with lower activation of the left and right amygdala. Left amygdala activation was positively correlated with mean alcohol craving (*r*_Left Amygdala_ = 0.52, *p* = 0.009, *p*_FDR_ = 0.01, 95% BCa CI = 0.17 to 0.78). Similarly, right amygdala activation was positively correlated with mean alcohol craving during the fMRI session (*r*_right Amygdala_ = 0.52, *p* = 0.010, *p*_FDR_ = 0.01, 95% BCa Cl = 0.16 to 0.80), suggesting that higher left and right amygdala activation was associated with higher subjective alcohol craving during the fMRI cue-reactivity paradigm. These findings suggest that increased CBD plasma levels are linked to decreased amygdala activation, while higher amygdala activation is associated with greater alcohol craving.

### Safety

Overall, no adverse or serious adverse events occurred after intake of a single dose of 800 mg CBD or placebo during assessment day.

## Discussion

The primary aim of the study was to compare the effects of a single dose of 800 mg oral CBD against matched oral PLC on amygdala response to emotional stimuli during a validated emotion processing fMRI paradigm and to explore associations between amygdala response, subjective alcohol craving and CBD plasma levels. The results of this neuroimaging RCT demonstrated that CBD significantly and specifically attenuated amygdala response to emotional stimuli, while response to neutral geometric shapes was unaffected. Higher amygdala activation across both groups significantly correlated with higher subjective alcohol craving, substantiating the link between neural responses to negative emotional stimuli and alcohol craving. Additionally, we observed a significant negative association between CBD plasma level and amygdala response to emotional stimuli, suggesting a plasma-level response association for the effects of CBD on amygdala activation.

These findings align with previous research indicating CBD’s effects on emotion processing, negative affect and amygdala reactivity both in healthy individuals (Fusar-Poli et al. 2010; Bhattacharyya et al. 2010) and in mental disorders (Crippa et al. 2011). Studies in social anxiety showed that CBD attenuated amygdala activation, which was associated with decreased anxiety symptoms and lower arousal (Fliegel and Lichenstein 2022). Similarly, a randomized-controlled trial in participants at high risk for psychosis showed that administration of a single oral dose of 600 mg CBD altered neural responses to emotional stimuli in the amygdala and other areas of the emotion processing neurocircuit (Davies et al. 2020). Thus, the results of the present trial are in line with prior evidence that CBD administration reduces amygdala response to negative emotional stimuli. Furthermore, our trial showed that amygdala attenuation was associated with lower alcohol craving, confirming the well-documented link between addiction, negative affect and craving and compatible with the concept of addiction as goal-directed drug choice that is driven by negative affective states (Hogarth 2020; Garrison et al. 2023; Childress et al. 1999). For CBD in AUD, preclinical studies have already suggested that CBD attenuates cue- and stress induced alcohol craving and alcohol consumption in animals (Turna et al. 2019; Viudez-Martínez et al. 2018; Nona et al. 2019). Our trial is the first RCT to demonstrate CBD’s effects in individuals with AUD, contributing to further understanding of the underlying neural mechanisms of CBD’s effects on emotion processing and AUD symptoms. For other substances, an anxiolytic and anti-craving effect of CBD in individuals with substance use disorders was already shown, with a previous RCT reporting a reduction of cue-induced craving and anxiety in individuals with opioid use disorder after oral CBD administration (Hurd et al. 2019). The RCT used doses of 400 mg and 800 mg CBD, demonstrating both acute effects shortly after CBD administration and protracted effects after repeated CBD administration (Hurd et al. 2019). In our trial, CBD’s effects on processing negative emotional stimuli were detected acutely after single dose intake of CBD and were associated with CBD plasma levels. CBD had no significant effects on task performance during the face-matching fMRI task, i.e. both groups showed similar response times and accuracy. A summary of behavioral performance measures during the emotion-processing task is presented in Table 1 (page 23). These findings align with previous studies reporting no effects of CBD on processing speed and attention (Hurd et al. 2019) and suggest that CBD does not impair processing of emotional stimuli, but instead might attenuate resulting negative affective states.

In summary, CBD versus PLC significantly attenuated bilateral amygdala reactivity to negative emotional stimuli, which was associated with CBD plasma levels, indicating that CBD attenuated amygdala activation in a plasma level-dependent manner. The significant association between amygdala activation and alcohol craving emphasizes the link between emotion processing in the amygdala and craving pathology in AUD. It might also indicate that CBD attenuates craving responses during negative affective states – in part – via its effects of emotion processing in the amygdala. Previous trials by our research group demonstrated that attenuation of amygdala activation through a distinct pharmacological agens, oxytocin, led to an attenuated amygdala’s response to negative emotional stimuli during face-matching task and confirmed the close association between amygdala activation and alcohol craving (Bach et al. 2021). Furthermore, prior research suggested that amygdala response to emotional stimuli may be used as a (bio-) marker for effectiveness of future pharmacological AUD treatments (Gowin et al. 2016) and for other substance use disorders (Li et al. 2015; Funk et al. 2016). For AUD, the current RCT is the first to establish the association between CBD, amygdala activation, alcohol craving and CBD plasma levels in humans. These findings provide a foundation for further exploring CBD’s effects and its potential as novel treatment for AUD.

## Limitations

The RCT was designed as first proof-of-principle study in AUD. Even though results support the robustness of presented findings, future trials are needed to confirm the generalizability and replicability of the results with extended focus both on acute and protracted effects of CBD. As the sample size was powered for the primary outcome, the present analyses are subject to reduced statistical power. Consequently, the study was sufficiently powered to detect large effects, whereas smaller or more subtle effects may have gone undetected. Nonetheless, the sample size is consistent with that of previous fMRI studies of comparable scope (Hurzeler et al. 2024). Another limitation might be found in a potential ceiling effect in task performance which might have masked subtle differences with overall high performance. Nevertheless, our findings are consistent with previous studies showing no impairing effects of CBD on task performances in processing or reaction time in individuals with substance use disorders (Hurd et al. 2019; Rizkallah et al. 2022; Batalla et al. 2020). Our trial used a single dose of CBD as it aimed to provide initial proof-of-concept for its effects on AUD patients, building on preclinical AUD evidence (Nona et al. 2019) and clinical evidence from other substance use disorders (Hurd et al. 2019) showing effects after a single administration. This design cannot assess long-term effects; however, existing data suggest that CBD’s benefits may persist even after discontinuation, justifying the focus on acute dosing in this early-phase trial (Hurd et al. 2019; Nona et al. 2019). With respect to data analysis, it should be noted that our approach was less stringent than the whole-brain correction of fMRI activation, but still seems reasonable with regard to the strong a priori hypothesis on the amygdala as regions of interest and prior evidence substantiating the central role of the amygdala in emotion processing and the applied face-matching paradigm. In addition, we followed established recommendations on performing small volume correction, e.g. the ROIs were determined independently of the specific test on which the correction is performed, using standardized anatomical masks (Poldrack et al. 2008).

## Conclusion

CBD attenuated the amygdala response to negative emotional stimuli in individuals with AUD. This effect was closely associated with CBD plasma levels and correlated with lower alcohol craving. These findings elucidate the neurobiological basis of CBD’s effects in AUD and indicate CBD’s potential effects on attenuating negative affective states and resulting craving in AUD and thus lay ground for further exploring CBD’s potential as novel treatment for AUD.

## Supplementary Information

Below is the link to the electronic supplementary material.

Supplementary file1 (DOCX 232 KB)

## Data Availability

The datasets generated and analyzed during the current study are available from the corresponding author upon reasonable request.
