# Supplementary material for: CBD attenuates amygdala response to negative emotional stimuli in individuals with alcohol use disorder – a randomized controlled trial
Source: Psychopharmacology (Berl). 2025 Aug 29;243(3):553–63. doi: 10.1007/s00213-025-06860-5 (PMC12979320; doi:10.1007/s00213-025-06860-5)
Supplement: Supplementary file 1 — Supplementary file1 (DOCX 232 KB) [file 213_2025_6860_MOESM1_ESM.docx]

**Supplementary Information**

**CBD attenuates amygdala response to negative emotional stimuli in individuals with Alcohol Use Disorder –**

**a randomized controlled trial**

**Content:**

- **Supplementary Methods**
- **Supplementary Table S1:** List of brain areas that show a significant higher activation during the presentation of negative emotional face stimuli compared to the presentation of neutral shape stimuli during the face-matching functional magnetic resonance imaging paradigm
- **Supplementary Figure S1:** Bar charts depicting the activation in the left and right amygdala during the different task contrast conditions

**Supplementary Methods**

**Sample Size Estimations**

The study was designed to detect at least medium-sized effects (f ≥ 0.25) of CBD on the **primary outcome**, i.e. brain activation in the nucleus accumbens in response to alcohol-associated visual stimuli during the alcohol cue-reactivity task, with a statistical power of ≥80%. Further methodological details and results of the alcohol cue-reactivity task have been published previously [1]. A priori sample size estimations indicated that n = 13 patients per group were needed to achieve 80% power for a repeated-measures ANOVA with twelve assessments, testing the main effect of treatment group (f ≥ .25, α = .05, two-sided, r among repeated assessments = .11). Conservative post-hoc sensitivity analyses for outcomes with only one repated assessment indicated a power of >80% to detect large effects (≥ 1.01) with the achieved sample size of n = 25 for the imaging analyses.

**Determination of Cannabidiol plasma levels**

**Blood samples**

10 ml blood plasma samples were taken via venuous puncture 210 minutes after Cannabidiol (CBD) administration and 10 minutes prior to the combined stress- and alcohol-cue-experiment. Blood samples were labeled with a Centraxx ID and date/ time of collection. They were transported to the local biobank, where they were centrifuged at 4000 rounds per minute (rpm). Plasma aliquots were transferred to new tubes, stored at – 80°C and sent for batch analysis at a specialized laboratory (MVZ Labor Dessau GmbH, Dessau, Germany) to reduce inter-assay variation.

**Blood sample preparation**

200 µL MeOH/ACN (50/50, v/v) containing 2 ng/mL CBD-d3 was added to 20 µL of plasma or control samples The mixture was vortexed and centrifuged at 13000 rpm for 5 min. Subsequently 55 µL of the supernatant was transferred to a 96-well plate and concentrated into 10 µL ethylene glycol at 60 °C in a vacuum evaporation centrifuge. The residue was mixed with 15 µL of mobile phase A (20 mM ammonium formate, 0.1% formic acid) and 2 µL was injected into the UPLC system.

**UPLC-MS/MS analysis**

CBD was quantified using an accredited routine UPLC-MS/MS method. Data were acquired with a Waters® Acquity® UPLC® connected to a Xevo® TQ-XS detector with an UniSpray™ ion source (Waters®, Eschborn). The detector operated in the positive ionization mode. Chromatographic separation was performed at 60 °C on a Waters 2.1 mm x 150 mm, 1.7 µm, BEH Phenyl column with pre-filter. Mobile phase A consisted of 20 mM ammonium formate plus 0.1% formic acid (pH 3.0) while mobile phase B was 0.1% formic acid in methanol. Gradient separation was performed within 11 min at a flow rate of 0.3 mL/min. The gradient program initiated with 10% mobile phase B, increased to 100% at 10 minutes and remained for 0.5 minutes before re-equilibration. During data acquisition, 0.0037% HCl was infused post-column with a flow rate of 5 µL/min to enhance ionization. The autosampler temperature was maintained at 8 °C.

Quantification was based on the response ratio of the target ion and the corresponding deuterated internal standard. Ten concentrations for CBD calibrators were prepared in EDTA whole blood ranging from 0.5 ng/mL to 120 ng/mL. The coefficient of determination (r2) was ≥ 0.99. Samples with concentrations exceeding 120 ng/mL were diluted 1:10 with PBS and re-analysed.

The method was validated following the GTFCh validation guidelines for quality assurance in forensic-toxicological analysis. Parameters evaluated include selectivity, linearity, limit of detection and quantification, precision, accuracy, matrix effect and stability. Full validation was performed using EDTA whole blood and the method was also cross-validated for plasma. Limit of detection (LOD) and limit of quantification (LOQ) were 0.3 ng/mL and 0.8 ng/mL, respectively.

**Supplementary Tables**

**Supplementary Table S 1**. List of brain areas that show a significant higher activation during the presentation of negative emotional face stimuli compared to the presentation of neutral shape stimuli during the face-matching functional magnetic resonance imaging paradigm (One sample t-test in SPM12, considering all *n* = 25 participants contrast “faces – shapes”, all results cluster-level whole-brain corrected at p_FWE_ < .05 and p_FWE_ < .05 small volume corrected for the Amygdala as pre-specified region of interest).

| **Side** | **Lobe** | **Brain areas** | **Cluster size (voxel)** | **MNI coordinates**  **(x, y, z)** | | | ***t*_max_** |
| --- | --- | --- | --- | --- | --- | --- | --- |
| *Faces > Shapes* | | |  |  |  |  |  |
| L  R  L  L  R  L  R | Temporal, Occipital  Temporal  Occipital | Inferior Temporal Gyrus, Fusiform Gyrus, Inferior/Middle/Superior Occipital Gyrus, Calcarine Gyrus  Inferior Temporal Lobe, Fusiform Gyrus, Inferior/Middle/Superior Occipital Gyrus, Calcarine Gyrus, Cerebellum  Thalamus, Hippocampus  Amygdala, Hippocampus  Parahippocampal Gyrus, Hippocampus, Amygdala, Thalamus  Hippocampus  Calcarine Gyrus | 2922  3437  160  275  607  34  1 | -24  26  -22  -22  20  -36  26 | -96  -94  -30  -8  -4  -26  -68 | 4  4  -2  -14  -14  -12  12 | 15.96  14.28  9.68  9.35  9.34  7.97  6.54 |
|  |  |  |  |  |  |  |  |

MNI = Montreal Neurological Institute

**Supplementary Figures**

**Supplementary Figure S1.** Bar charts depicting the activation in the left and right amygdala during the different task contrast conditions (face > baseline (bsl), shapes/forms > baseline, face > shapes/forms) illustrating that the effects of CBD was specific for the presentation of emotional stimuli.


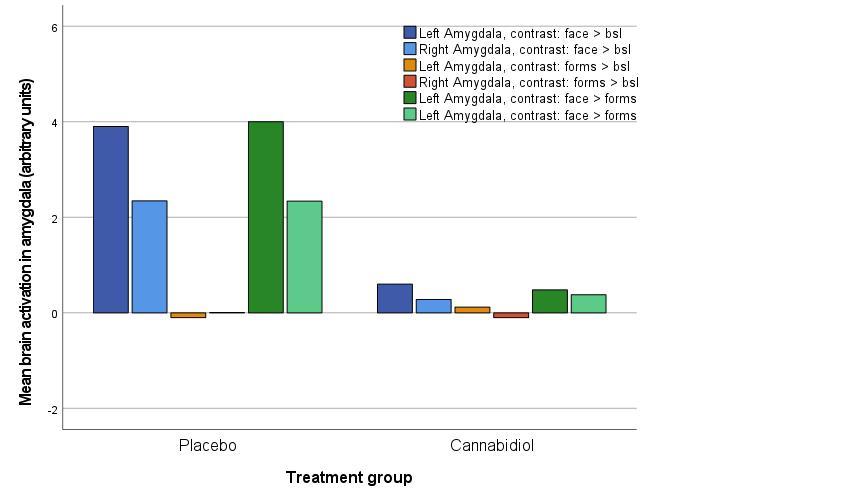


**Supplementary Figure S2. CONSORT study flow chart**


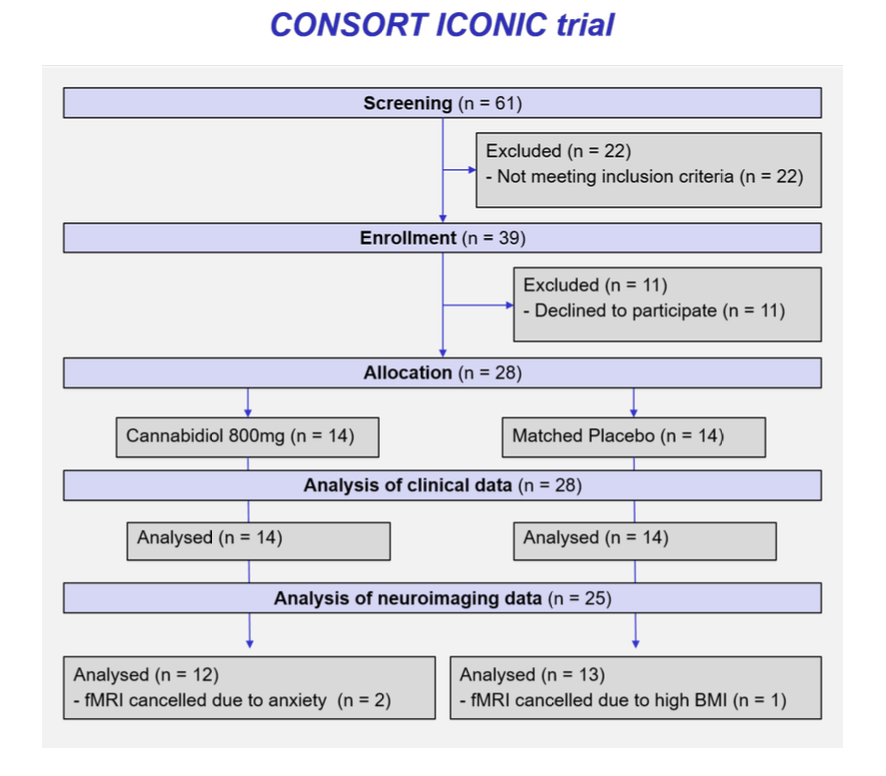


1. Zimmermann, S., et al., *Acute cannabidiol administration reduces alcohol craving and cue-induced nucleus accumbens activation in individuals with alcohol use disorder: the double-blind randomized controlled ICONIC trial.* Mol Psychiatry, 2024.
